# Structure‐Activity Relationship of Defective Metal‐Based Photocatalysts for Water Splitting: Experimental and Theoretical Perspectives

**DOI:** 10.1002/advs.201900053

**Published:** 2019-03-22

**Authors:** Yong‐Chao Zhang, Nisha Afzal, Lun Pan, Xiangwen Zhang, Ji‐Jun Zou

**Affiliations:** ^1^ Key Laboratory for Green Chemical Technology of Ministry of Education School of Chemical Engineering and Technology Tianjin University Tianjin 300072 China; ^2^ Collaborative Innovative Center of Chemical Science and Engineering (Tianjin) Tianjin 300072 China

**Keywords:** defect engineering, defects‐activity relationship, metal‐based materials, photocatalysis, water splitting

## Abstract

Photocatalytic water splitting is promising for hydrogen energy production using solar energy and developing highly efficient photocatalysts is challenging. Defect engineering is proved to be a very useful strategy to promote the photocatalytic performance of metal‐based photocatalysts, however, the vital role of defects is still ambiguous. This work comprehensively reviews point defective metal‐based photocatalysts for water splitting, focusing on understanding the defects' disorder effect on optical adsorption, charge separation and migration, and surface reaction. The controllable synthesis and tuning strategies of defective structure to improve the photocatalytic performance are summarized, then the characterization techniques and density functional theory calculations are discussed to unveil the defect structure, and analyze the defects induced electronic structure change of catalysts and its ultimate effect on the photocatalytic activity at the molecular level. Finally, the challenge in developing more efficient defective metal‐based photocatalysts is outlined. This work may help further the understanding of the fundamental role of defect structure in the photocatalytic reaction process and guide the rational design and fabrication of highly efficient and low‐cost photocatalysts.

## 1 Introduction

Conversion of solar energy to renewable energy like hydrogen energy is a feasible, sustainable, and promising method to reduce the dependence on fossil fuels to meet energy demands and environmental concerns.^1^, ^2^, ^3^, ^4^ As a simple and effective method for hydrogen production, water splitting (2H_2_O → 2H_2_ + O_2_) is very prospective for solar energy storage and utilization.^1^, ^3^ However, photocatalytic water splitting is an uphill reaction and faces with excessive limitations such as low solar efficiency, high‐cost, and catalysts photocorrosion etc. Until now, many materials have been developed that are prospective for photocatalytic water splitting or the half‐reactions of hydrogen evolution (2H^+^ + 2e^−^ → H_2_) and oxygen evolution (2H_2_O + 4h^+^ → 4H^+^ + O_2_) with the assistance of sacrificial agents.^3^, ^5^, ^6^ Metal‐based catalysts including metal oxides of TiO_2_, Co_3_O_4_, CeO_2_, and ternary compounds materials of BiVO_4_, BiO*_m_*X*_n_* (X = Cl, Br, and I) as single photocatalyst systems have attained excessive attention due to their relatively high activity.^5^, ^7^, ^8^, ^9^, ^10^, ^11^ However, these photocatalysts are still far away for applications because of their low efficiency (due to the unsuitable energy band values and position, rapid recombination of photogenerated holes and electrons, and sluggish surface reaction kinetic) and relatively high‐cost. Therefore, there is vital need to design and fabricate more efficient photocatalysts.

Several strategies have been developed to optimize photocatalysts, including phase and morphology control, crystal facet and defect engineering, microelectronic structure modulation, construction of homojunction and heterojunction, and many remarkable results have been achieved.^3^, ^5^, ^10^, ^12^ Among them, defect engineering is an effective method to regulate the atom coordination number and electronic structure, to improve the mobility of carriers and conductivity, to tune surface properties including the interaction between vacancies sites, reactants or intermediates, and therefore to improve the activity of photocatalysts, as confirmed by recent experimental studies and theoretical calculations.^5^, ^7^, ^10^, ^13^, ^14^, ^15^ In particular, 0D point defects are most common and easy to synthesize than 1D line defects, 2D planar defects, and 3D volume defects.^16^, ^17^ The conductivity of most semiconductor materials is attributed to the nonstoichiometric composition caused by point defects. However, line defects and planar defects do not change or seldom change the stoichiometric compositions.^17^ An in‐depth understanding of the role of point defects also helps to studying line defects, planar defects, and even volume defects.^18^ Chen et al. synthesized disordered TiO_2_ nanocrystals under harsh hydrogen conditions, and found that the optical absorption enhancement and energy band structure changes with appearance of mid‐gap states and conduction band (CB) tail states caused by disorder in the surface layer, which can greatly improve the photocatalytic H_2_ generation under full wavelength light.^14^ Li et al. found the presence of anion vacancies in BiOCl significantly improves the photocatalytic activity, attributed to the fact that surface oxygen vacancies accelerate the photocatalytic reaction kinetics and change the related photocatalytic mechanism.^10^ P‐doped CdS with rich sulfur vacancies is effective for water splitting without sacrificial agents, attributed to the synergy between sulfur vacancies and phosphorus dopant.^19^ For BiVO_4_, the incorporation of oxygen vacancies can overcome the problem of poor activity and conductivity, and greatly enhance photocatalytic water oxidation.^9^ In addition, semiconductors like TiO_2_, ZnO, and Co_3_O_4_ with abundant metal defects and excellent photocatalytic properties have been reported.^5^, ^7^, ^8^, ^13^, ^20^, ^21^ Moreover, quantum chemistry simulation and advanced characterization technologies have been used for deep understanding of microstructure, reactive sites, and the structural‐activity characteristics of photocatalysts at the atomic level.

Some review about the role of defects in photocatalysts have been reported previously,^17^, ^22^, ^23^ however the intrinsic role of defect effects for photocatalytic water splitting is still an open question. In this review, typical defective metal‐based catalysts including metal oxides and ternary compound semiconductors with anion and cation vacancies or simultaneously containing both anion and cation vacancies for photocatalytic water splitting are summarized. Further, recent breakthrough in synthetic methods as well as strategies to regulate defects and corresponding characterization methods are discussed. Moreover, the effect of defects in photocatalytic process is systemically analyzed. Then based on experimental results and theoretical calculations, the water splitting mechanism and the relationship between defects and reactivity was discussed. Last, the challenges in current technology and future development of defective catalysts in photocatalytic water splitting are prospected.

## 2 Types of Defect in Photocatalysts

Defect engineering without introducing other dopant element is a prevalent and useful method to improve the photocatalytic activity, as it can modulate the geometric and electronic structure of catalyst, thereby extending light harvesting, accelerating carrier separation and migration, and promoting surface reaction.^14^ Structurally disordered photocatalysts can form anion vacancies (typically oxygen vacancies and halogen vacancies), cation vacancies (metal vacancies) or both anion and cation vacancies. For example, various metal oxides including TiO_2_, BiO_2_, WO_3_ as well as some typical ternary compound semiconductors of BiVO_4_, BiOX (X = Cl, Br, and I) have attracted widespread attention for water splitting and half‐reaction of hydrogen or oxygen evolution because of their intrinsic high activity and stability.^5^, ^7^, ^10^, ^11^, ^24^ In this section, we summarize typical metal‐based photocatalysts to show the change of surface structure, electron, energy band, optics and redox reactions caused by different types of point defects in photocatalytic water splitting.

### 2.1 Anion Vacancies

Anion vacancies (such as oxygen, sulfur, and halogen vacancies) are common features of metal‐based catalysts due to the low formation energy, as evidenced by many experimental studies and theoretical calculations.^5^, ^25^, ^26^, ^27^ Generally, anion vacancies engineering can effectively regulate the electronic structure and energy band structure of photocatalysts, reduce atom coordination numbers and provide more active centers, which plays important role in improving photocatalytic efficiency.

**Figure 1:**
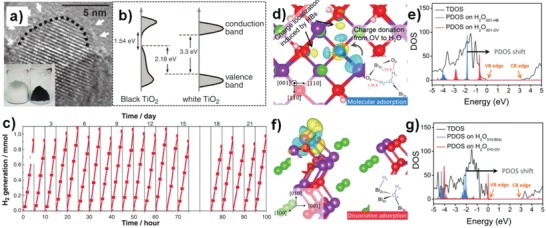
a) High resolution transmission electron microscopy (HRTEM) images of TiO_2_ nanocrystals after hydrogenation, and b) DOS of disorder‐engineered black TiO_2_ nanocrystals. c) The hydrogen evolution from water splitting on disorder‐engineered black TiO_2_ nanocrystals. Reproduced with permission.^14^ Copyright 2011, Science. d–g) The charge density difference of water‐adsorbed BiOCl surfaces and the corresponding PDOS of adsorbed water. Reproduced with permission.^32^ Copyright 2016, American Chemical Society.

As an important form of anion defects, oxygen vacancies have been extensively studied for photocatalytic water splitting.^5^ Chen et al. fabricated black TiO_2_ nanocrystals with surface layers disorder by hydrogenation treatment, which presented high activity and stability of sunlight‐driven hydrogen production with the presence of sacrificial reagent (**Figure** **1**a,b,c).^14^ Zhang et al. synthesized surface‐ and bulk‐rich oxygen‐defective TiO_2_(001), and found surface oxygen defects can effectively regulate the conduction band position along with inducing a band tail located above the reduction potential of H^+^/H_2_ and promote photocatalytic hydrogen generation. By contrast, bulk oxygen vacancies in TiO_2_ suppress the hydrogen production because the defect‐induced conduction band location is lower than the reduction potential of H^+^/H_2_.^28^ Wang et al. fabricated oxygen vacancies tungsten trioxide by thermal treatment with controlling hydrogen concentration, and found both surface and bulk oxygen vacancies can promote photocatalytic water splitting, but with different mechanisms.^29^ Surface oxygen vacancies primarily lowers the valence band (VB) edge to enhance photogenerated carriers separation, while bulk oxygen vacancies narrows the bandgap energy to promote visible light harvesting and slightly inhibit carriers recombination. Penfold et al. demonstrated that photogenerated holes can be trapped at oxygen vacancies sites in ZnO by using X‐ray absorption and dispersive X‐ray emission spectroscopy.^30^ Yu et al. found that large amounts of surface oxygen defects on BiVO_4_ are not conducive to photocatalytic oxygen evolution because the defects may act as recombination centers of photogenerated carriers.^31^ Li et al. reported oxygen vacancies in BiOCl can effectively regulate the geometric and electronic structures of adsorbed water, and more localized electrons transfer from oxygen vacancies sites to the adsorbed water, which in turn promotes the photocatalytic activity of water oxidation (Figure 1d,e,f,g).^10^, ^32^

Other anion vacancies in photocatalysts, such as halogens and sulfur vacancies have also been reported. Wang et al. synthesized ZnS with controlled sulfur vacancies concentration by using different amount of reducing agent, and found the photocatalytic hydrogen production activity increases until suitable sulfur vacancies content is achieved.^33^ Zhang et al. found that surface sulfur vacancies in Zn‐Cd‐S solid solution induces a narrow bandgap and better photocatalytic performance, which was also confirmed by density functional theory (DFT) calculation.^34^ Surface sulfur defects facilitate the migration of charge carriers from bulk phase to the surface, which is beneficial for photoreaction. For example, the hydrogen production rate of sulfur‐deficient photocatalyst under visible light irradiation is much higher than sample without surface defects. Zhang et al. reported that surface/subsurface oxygen defects of ultrathin BiOCl nanosheets can achieve solar‐driven water splitting without sacrificial agent and cocatalysts.^35^ Surface oxygen defects can effectively regulate the position of the energy band, yielding mid gap states or band tail states (Figure 1b), increase carrier density, and promote the separation and migration of carriers. However, for CdS with surface sulfur defects, photogenerated carriers are trapped due to defects and redox reactions occurring on the surface, which results in severe photocorrosion.^6^

Remarkably, anion vacancies can improve the photoresponsive ability of photocatalyst by adjusting energy band structure. The presence of anion vacancies can create mid gap states and narrow bandgap in semiconductors to promote light harvesting, and therefore photocatalysts display obvious color change. Oxygen‐deficient anatase is blue compared to the perfect white counterpart, and TiO_2_ photocatalyst induced by atomic hydrogen‐occupied oxygen vacancies shows red color with strong visible light absorption capability.^36^ The oxygen defective WO_3_ exhibits a deep blue color attributed to intervalence charge transitions compared to pristine WO_3_ (light blue).^26^ Oxygen‐vacancy‐enriched ZnO shows yellow color, while powdery ZnO shows white color.^37^ The color of BiOI*x* changes from dark red to orange as the iodine content decreases.^38^

### 2.2 Cation Vacancies

Cation vacancies acting as shallow acceptors can induce p‐type conductivity, enhance the migration of holes, and play an important role in photocatalytic process, as reported by many experimental studies and theoretical calculations.^7^, ^8^, ^20^ The presence of cationic defects gives many novel properties to photocatalysts, for example, regulating band structure by upward shift in the VB maximum and a downward shift in the CB minimum without new middle states formation, and promoting separation and rapid migration of photogenerated carriers. However, the difficulty in catalyst synthesis due to the high formation energy as well as the structure instability caused by the cation defects still faces huge challenges.

**Figure 2:**
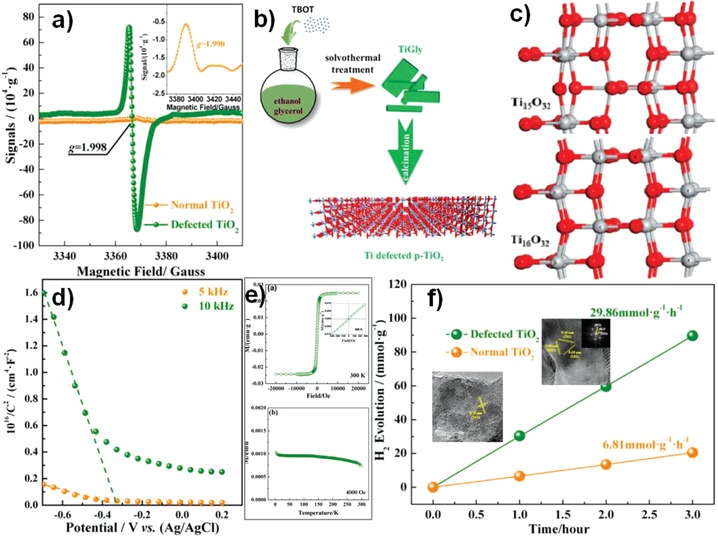
a) Low temperature (120 K) EPR spectra of defected and normal TiO_2_. b) Schematic fabrication procedures of Ti defected TiO_2_ and c) optimized cell structures of Ti‐defected TiO_2_ and normal anatase TiO_2_. d) Mott–Schottky plots measured in a standard three‐electrode setup using defected TiO_2_ electrode as working electrode. e) Magnetization (*M*–*H*) curves of defected TiO_2_ measured at 300 K and *M*–*T* curve of defected TiO_2_ in the field of 4000 oersted. f) Time course of hydrogen generation. Reproduced with permission.^7^ Copyright 2015, American Chemical Society.

Our group developed a series of stable cation‐defective catalysts of TiO_2_, ZnO, Co_3_O_4_, and Mn*_x_*Co_3_ *_−x_*O_4_ through simple solvothermal and calcination methods.^7^, ^8^, ^20^, ^39^ Anatase TiO_2_ with metal vacancies exhibits many novel physicochemical properties, for example, nonferromagnetic n‐type TiO_2_ is converted to p‐type TiO_2_ with room temperature ferromagnetism (**Figure** **2**d). Moreover, Ti‐defective TiO_2_ shows high photocatalytic hydrogen evolution performance due to the improvement in carrier separation and migration efficiency, especially improvement of holes migration (Figure 2f).^7^ The presence of Zn defect sites in ZnO also produced properties similar to Ti‐defective TiO_2_, such as p‐type conductivity, room temperature ferromagnetism, and high photocatalytic activity.^20^ Meanwhile, zinc vacancies introduced in ZnS via hydrothermal reaction and sulfide addition as sulfur source not only effectively protects the catalyst from photocorrosion with high photostability by increasing valence band position to reduce the oxidizing ability of photogenerated holes, but also promotes photocatalytic hydrogen evolution activity due to the enhancement of charge separation and electron transfer.^40^

We also synthesized Co_3_O_4_ with rich cobalt defects, and the apparent electron delocalization accompanying cation vacancy improves the conductivity, increases the number of surface active sites, and facilitates the activation of water molecules, which leads to high oxygen evolution activity.^8^ Furthermore, Mn*_x_*Co_3_ *_−x_*O_4_ containing both manganese vacancies and cobalt vacancies shows high oxygen reduction reaction activity attributed to the enhanced conductivity and O_2_ adsorption ability.^39^ Zhao et al. demonstrated that nickel vacancies in NiO increase the conductivity and promote electron transfer ability during electrocatalysis.^41^ Fu et al. found that indium vacancies in In_2_S_3_ play a key role in improving photocatalytic hydrogen evolution, while pristine In_2_S_3_ shows no activity.^42^ For ternary compound semiconductors, the presence of cationic vacancies also improves photocatalytic water splitting. Apart from oxygen vacancies, Bi vacancies also exist widely in nonstoichiometric bismuth‐oxide compounds. Guan et al. synthesized ultrathin BiOCl nanosheets with triple vacancy associates V_Bi_″′V_O_V_Bi_″′, which enhances the light absorption, accelerates the separation of charge carriers, and significantly improves solar‐driven photocatalytic activity.^43^

### 2.3 Coexistence of Anion and Cation Vacancies

In addition to single type of defect structure, metal‐based defective photocatalysts containing both anion and cation vacancies have also been developed. The anion and cation vacancies coexist in one photocatalyst is a promising way to improve charge separation by forming a built‐in electrical field and the photocatalytic activity is increased. Especially, p–n homojunction is formed by materials with identical composition and/or crystal structure can provide continuity of band bonding and accelerate the charge transfer across the interface more efficiently.

**Figure 3:**
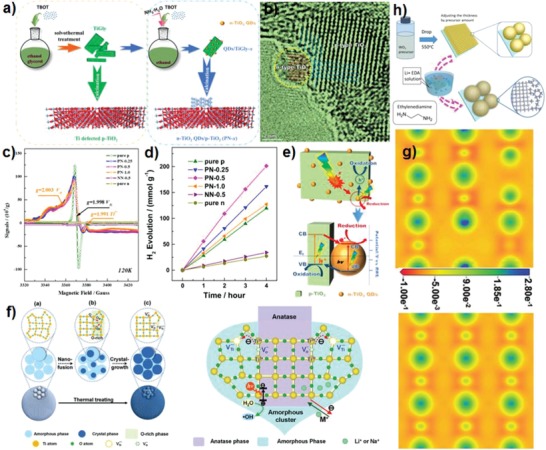
a) Schematic fabrication procedures of p‐type TiO_2_ and TiO_2_ p–n homojunction. b) TEM images. c) Low‐temperature (120 K) electron paramagnetic resonance (EPR) spectra of p‐type TiO_2_, n‐type TiO_2_, PN‐*x*, and NN‐0.5. d) Time course of photocatalytic hydrogen generation. e) Schematic illustration of charge separation and transfer in p–n homojunction. Reproduced with permission.^13^ Copyright 2016, Elsevier. f) The illustration of the formation of the amorphous‐anatase TiO_2_ from amorphous TiO_2_ and proposed mechanism of the photocatalytic properties. g) Charge density difference of TiO_2_ with junction of O‐vacancies and Ti‐vacancies and normal TiO_2_. Reproduced with permission.^46^ Copyright 2018, Wiley‐VCH. h) Schematic diagram of the fabrication procedure for Li‐EDA treated WO_3_. Reproduced with permission.^47^ Copyright 2016, Wiley‐VCH.

Using typical p‐type TiO_2_ with titanium vacancies as substrate, our group deposited n‐type oxygen vacancies TiO_2_ nanoparticles (**Figure** **3**a). The p–n homojunction TiO_2_ presents much higher performance for photocatalytic hydrogen evolution than p‐TiO_2_, owing to the improved carrier separation and migration efficiency with greater driving force (Figure 3d).^13^ In this system, titanium vacancies are shallow acceptors (p‐type conductivity) and oxygen vacancies are shallow donors (n‐type conductivity), and the formed internal electrical field in p–n homojunction TiO_2_ accelerate interface charge transfer (Figure 3e).^44^, ^45^ Similarly, we also synthesized ZnO p–n homojunction by depositing n‐type oxygen vacancies ZnO nanoparticles on the surface of p‐type metal vacancies ZnO, which exhibits high‐performance in photo‐electrochemical water splitting.^21^ Wu et al. synthesized homojunction of oxygen and titanium vacancies amorphous‐anatase homogeneous TiO_2_ by directly decorating interfacial titanium vacancies p‐type TiO_2_ around oxygen vacancies n‐type TiO_2_ nanocrystals (Figure 3f).^46^ The presence of oxygen defects causes the O and Ti atoms around oxygen vacancies sites to get more electrons. The neighboring O and Ti atoms of titanium vacancies sites get less electrons (Figure 3g). Therefore, the unique electron pathway from interfacial n‐type to p‐type facilitates the mobility of charge carriers. Moreover, around 93% of photocatalytic activity is retained after five cycles of photocatalysis, and oxygen vacancies and Ti vacancies signal from electron spin resonance (ESR) and ^1^H triple‐quantum and single‐quantum magic‐angle spinning nuclear magnetic resonance (^1^H TQ‐SQ MAS NMR) spectra respectively are still strong, suggesting the homojunction of oxygen vacancies and titanium vacancies TiO_2_ has high stability. WO_3_ photoanodes with both oxygen and tungsten vacancies show high photo‐electrochemical water oxidation activity due to the increased charge density and improved carrier transfer efficiency. The dual‐vacancy cover layer effectively suppress the rapid transport and accumulation of holes, thereby preventing the formation of peroxide species during water oxidation, therefore the catalysts possess high stability.^47^ As shown in Figure 3h, the mesoporous WO_3_ containing oxygen and tungsten vacancies is synthesized by using reducing solution of lithium ethylenediamine, and the over layer structure imparts positive role to enhance water oxidation activity. Zhu et al. found that bismuth vacancies and oxygen vacancies can be formed in the bulk of BiPO_4_ simultaneously to ensure the charge balance with bandgap narrowing during the ball‐milling process, but bulk defects inhibit the separation of carriers and corresponds to poor photocatalytic activity.^48^ Metal‐based photocatalysts containing both anion and cation vacancies have some novel properties that may be a promising photocatalyst.

## 3 Fabrication and Characterizations of Defects

### 3.1 Controllable Fabrication of Defects

Accurate synthesis of defective metal‐based catalysts with specific defect types, defect locations, and defect concentrations is of great significance for tuning surface electronic structure and valence binding energies of intermediates, and activity of photocatalytic water splitting. However, in most case, defects types, defects location and defects concentration of photo‐catalysts are difficult to control during synthesis.^49^ **Table** **1** summarizes typical defective metal‐based catalysts with different synthesis methods. For generation of anion defects, hydrogen heat treatment (such as high temperature hydrogen reduction), in situ reduction by chemical reducing agents (such as NaBH_4_, KBH_4_, N_2_H_4_, NaH, hydrazine, ethylene glycol, methanol, Zn powder, and Al powder), and photoreduction are useful strategies.^5^, ^26^, ^29^, ^50^, ^51^, ^52^ Significantly, there are three steps in the hydrogen reduction process, briefly, step 1: hydrogen interaction with the lattice anion. Step 2: electrons transfer from the adsorbed hydrogen to anion. Step 3: surface lattice anion is extracted by hydrogen to form H_2_X (X = anion) and leads to the formation of anion vacancies. Mostly, post‐treatment of catalysts using surface reduction can remove the surface anion to form surface anion vacancies. However, hydrogen reduction can easily generate bulk defects due to easy of hydrogen diffusion, and some studies show the bulk anion defects usually act as carrier recombination sites, which is not conductive to reactions.^50^ In contrast, some studies found that both surface and bulk anion defects play positive role in improving photocatalytic activity.^29^ Wang et al. found that during thermal treatment the bulk oxygen vacancies in WO_3_ increase with hydrogen concentration, but the surface oxygen vacancies concentration presents a volcanic curve change.^29^ Oxygen vacancies concentration in TiO_2_ can be tuned by controlling the amount of zinc powder reducing agent. The more the reducing agent, the higher the oxygen deficiency concentration, but excessive oxygen defects cause a decrease in photocatalytic performance, because proper amounts of Zn powder can completely convert TiO_2_ *_−x_* nanocrystals into highly active rutile phase.^53^

**Table 1:** Summary of typical defective metal‐based photocatalysts with different synthesis methods

Catalysts	Defect manufacturing method	Defect type	Application	Ref.
NiCo_2_O_4_, ZnCo_2_O_4_, Co_3_O_4_	Heat treatment under air atmosphere	Oxygen vacancy	Electrocatalytic water oxidation	70
ZnS	Adding sulfur powder	Sulfur vacancy	Photocatalytic H_2_ evolution	71
TiO_2_	Annealing under ultrahigh vacuum and high temperature	Oxygen vacancy	Electrocatalytic hydrogen evolution	59
ZnO	High‐pressure torsion	Oxygen vacancy	RhB photodegradation	54
TiO_2_	Aluminum reduction	Oxygen vacancy	Sonic‐/photoinduced tumor eradication	51
ZnS	Hydrothermal with adding sodium sulfide	Zinc vacancy	Photocatalytic H_2_ evolution	40
TiO_2_	Vapor‐induced hydro‐thermal hydrolysis	Oxygen and titanium vacancies	Benzene photocatalytic oxidation	50
CeO_2_	NaBH_4_ hydrothermal reduction	Oxygen vacancy	Photocatalytic water oxidation	5
BiOCl	Redox reaction between BiOCl and hot ethylene glycol	Oxygen vacancy	Photocatalytic water oxidation	32
BiO_2−_ *_x_*	Hydrothermal and liquid exfoliation	Oxygen vacancy	RhB and phenol photodegradation	24
WO_3_	Solution‐based reducing agent	Oxygen and tungsten vacancies	Photo‐electrochemical water oxidation	47
FeO*_x_*	Calcining with different atmosphere and time	Oxygen vacancy	Nitroarene hydrogenation	27
Co_3_O_4_	Hydrothermal method with controlling crystalline	Oxygen vacancy	Supercapacitors	72
ZnO	Solvothermal with thermal calcination	Metal vacancy	RhB photodegradation	20
TiO_2_	Solvothermal with thermal calcination	Metal vacancy	Photocatalytic H_2_ evolution and organics degradation	7
Co_3_O_4_	Solvothermal with thermal calcination	Metal vacancy	Electrocatalytic oxygen evolution reaction	8
BiOCl	Alcohol method	Metal vacancy	RhB photodegradation	43
BiPO_4_	Ball‐milling method	Oxygen and metal vacancies	MB photocatalytic degradation	48
TiO_2_	Solvothermal with thermal calcination	Oxygen and metal vacancies	Photo‐electrochemical and photocatalytic H_2_ production	13
Fe–Mn–O hybrid nanosheets	Reflux and low‐temperature calcination	Oxygen and metal vacancies	Electrocatalytic water oxidation	73
W_18_O_49_	Molybdenum doping	Oxygen vacancy	Solar‐driven nitrogen fixation	74
WO_3_	Thermal treatment with H_2_	Oxygen vacancy	Visible light photocatalytic water oxidation	29

Apart from the above mentioned reduction methods, surface anion vacancies can be generated by using high‐pressure torsion (HPT) method,^54^ plasma techniques,^55^ atomic layer deposition (ALD),^56^ amorphous crystal growth,^57^ liquid exfoliation method thermal treatment,^24^ hydrothermal treatment with the assistance of transition metal,^58^ ultrahigh vacuum,^59^ and electrochemical and photoassisted methods.^60^, ^61^ Wang et al. reported a series of metal oxides rich in anion defects for electrocatalytic oxygen evolution reaction (OER) and hydrogen evolution reaction (HER) by using plasma etching methods in different atmospheres (such as N_2_, NH_3_, and Ar).^62^, ^63^ During the process of plasma‐engraving, metal‐based oxides were deposited on Ti substrate by using chemical vapor deposition or electrodeposition methods, and then subjected to plasma treatment with different pressure and treatment time. Remarkably, the plasma etching not only produces anion defects but also leads to high surface area, which ensures catalysts with more active sites and high electron conductivity. Nitrogen doping and oxygen defects can be achieved simultaneously in Co_3_O_4_ by N_2_ plasma.^64^ Vapor‐induced hydrolysis method can also produce nanocrystals with a suitable ratio of surface defects and bulk defects. BiOCl with in situ deposition of cationic Bi nanowires also causes the formation of oxygen vacancies.^65^ Large amount of oxygen vacancies in ZnO rocksalt phase can be produced by using HPT method at 6 GPa.^54^ Moreover the resulted reduced bandgap of ZnO significantly improves the photocatalytic activity, and this method is also applicable to perovskite oxides. ALD method can introduce oxygen vacancies on surface of TiO_2_ layer without affecting bulk properties, which promotes the adsorption and activation of N_2_, and facilitates the photocatalytic N_2_ reduction.^56^ As a matter of fact, more surface anion vacancies are formed during the post‐treatment in most cases, while bulk and surface vacancies can be simultaneously formed by in situ reduction during the synthesis of photocatalysts. Our previous study shows that oxygen vacancies in CeO_2_ by in situ reduction can form surface and bulk vacancies, and the presence of oxygen defects is favorable for photocatalytic water oxidation, and also have been confirmed by DFT calculations.^5^ However, most surface anion defects in metal‐based catalysts are metastable and easily repaired due to the trapping of anion species.^66^ Therefore, maintaining surface anion defects and preventing the self‐repairing to improve catalyst stability are necessary.

Despite great difficulty, cationic‐defective metal‐based catalysts can also be fabricated by controlled synthesis methods. We developed a promising method to obtain highly active and stable cationic‐deficient metal‐based catalysts (TiO_2_, ZnO, Co_3_O_4_, and MnCoO). Metal oxide precursor is mixed with glycerol to obtain a glycerol compound, subjected to a high‐temperature baking treatment which results in generation of oxygen‐rich structure (Figure 3a).^7^, ^8^, ^13^, ^20^, ^39^ Actually, the glycerol with metal precursor play critical role in forming metal defects. During the thermal calcination, the organic groups of glycerol precursor are removed and metal‐oxygen‐metal lattice chains couple with each other. The terminal oxygen atoms bind with surface metal atoms to form many metal voids. Therefore, a metal‐defective oxide is attained. However, a high calcination temperature above 500 °C will leads to recrystallization of catalysts and reduce the metal defects concentration.^8^ On the other hand, one‐step cation exchange can effectively synthesize cation defective Cu_2−_ *_x_*S nanowires.^67^ Hydrothermal method is developed to synthesize indium vacancies in In_2_S_3_. Adding other anionic salts can produce cationic vacancies.^40^ The hydrothermal hydrolysis temperatures, thermal annealing temperatures, times, atmospheres, particle size, and crystallinity can be controlled to obtain a catalyst with a specific concentration ratio of bulk defects to surface defects.^3^, ^28^ Wang and co‐workers synthesized highly efficient electrocatalysts with rich metal defects by using plasma etching.^68^, ^69^ The produced metal vacancies promote the exposure of more active sites, which is favorable for electrocatalytic activity enhancement.

### 3.2 Characterization Methods of Defects

Understanding the structure of defects by using characterizations help to unveil the relationship between structure and activity, which is highly important for designing of high‐performance defective catalysts.

**Figure 4:**
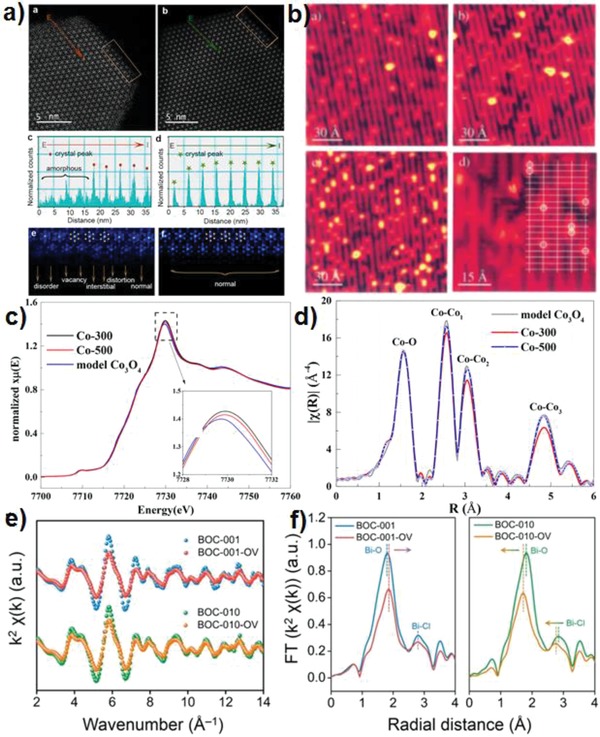
a) HAADF‐STEM images of KLu_2_F_7_:38%Yb^3+^, 2%Er^3+^ nanoparticles. Reproduced with permission.^75^ Copyright 2017, American Chemical Society. b) The H_2_O/TiO_2_(110) system at room and low temperature ((a') The bare surface imaged at room temperature. (b') After 10^−2^ L of water exposure at room temperature. (c') After exposure to 10^−2^ L of H_2_O at 180 K, imaged at 160 K. (d') Same conditions as (c')). Reproduced with permission.^77^ Copyright 2001, American Institute of Physics. c) XANES and d) Fourier transforms of k‐space oscillations for Co_3_O_4_, Co‐500, and Co‐300. Reproduced with permission.^8^ Copyright 2018, American Chemical Society. e) Bi L‐edge extended XAFS oscillation function *k*2χ(*k*) and f) the corresponding Fourier transforms for the BiOCl with and without OVs. Reproduced with permission.^32^ Copyright 2016, American Chemical Society.

Herein, some qualitative and quantitative defect characterization methods to identify and quantify defects in photocatalysts from a microscopic perspective are summarized. Electron microscopy technology provides the atomic structure of imaging materials. High‐resolution noncontact atomic force microscopy (NC‐AFM), high‐angle annular dark‐field scanning transmission electron microscopy (HAADF‐STEM), and high‐angle annular dark field (HAADF) microscopy are useful tools for studying the surface structure of materials, and are being widely used for structural characterization of defective catalysts. For instance, the surface structure of lanthanide‐doped KLu_2_F_7_ nanoparticles are clearly recognized by using HAADF‐STEM, and its edge structure can be characterized by the peak‐to‐valley intensities.^75^ Moreover, the uncompleted crystallization leads to the formation of lattice disorder and surface defect states (see from HAADF‐STEM characterization) (**Figure** **4**a). STEM can be used to distinguish surface and bulk defects.^76^ Light element sensitive annual bright field (ABF)‐STEM images also give evidence on surface structure deformation and defects of metal‐based catalysts directly. For example, at low coverage, water splitting only occurs at oxygen‐defective sites in defective TiO_2_. Scanning tunneling microscopy (STM) directly demonstrated the interaction between oxygen‐deficient sites and hydroxyl groups formed after water dissociation (Figure 4b).^77^

X‐ray absorption spectroscopy (XAS), including extended X‐ray absorption fine structure (EXAFS), and X‐ray absorption near‐edge structure (XANES) spectroscopy, is effective technique to determine the average oxidation state and coordination environment of elements, and the distortion of local lattice in materials. For example, Zhang et al. used XANES to determine the metal vacancies in Co_3_O_4_.^8^ As shown in Figure 4c, Co K‐edges for metal‐defective Co_3_O_4_ has higher energy than that of pristine Co_3_O_4_, which confirms that they possess higher oxidation state. R space is identified by Fourier transformation from wavevector *k*, which depends on the coordination number and mean‐square disorder. Defective Co_3_O_4_ has lower coordination number of Co–Co and mean‐square disorder, indicating more metal defects in the crystals (Figure 4d). Figure 4e shows the Bi L‐edge extended XAFS *k*2χ(*k*) oscillation curves of BiOCl, and the presence of oxygen vacancies remarkably lowers the amplitude but does not change the frequencies. The existence of oxygen vacancies in BiOCl is identified by quantitatively fitting the change in coordination number of the surface interatomic Bi–O pair and Bi–Cl pair (Figure 4f).^32^ Surface‐sensitive soft X‐ray techniques and bulk information‐included hard X‐ray techniques can be used to distinguish the surface and bulk defects. For example, using total electron yield (TEY) mode, O K‐edge XANES spectra can provide surface information of hydrogenated TiO_2_, whereas in fluorescence mode the Ti K‐edge XANES can give bulk information.^78^ Besides, in situ electron energy loss spectroscopy (EELS) is used to analyze the distribution of oxygen vacancies in reduced TiO_2_.^59^

**Figure 5:**
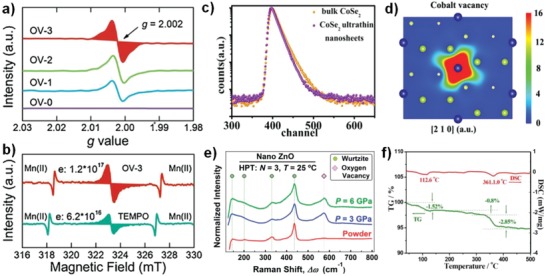
a) EPR spectra of different samples with the same weight (red area showed the integration method for OV quantification). b) Quantification of the oxygen vacancies concentration of sample (OV‐3). Reproduced with permission.^80^ Copyright 2018, Elsevier. c) Positron lifetime spectrum of ultrathin CoSe_2_ nanosheets and bulk CoSe_2_, respectively. d) Schematic representations of trapped positrons of cobalt vacancies. Reproduced with permission.^82^ Copyright 2014, American Chemical Society. e) Raman spectra of ZnO before/after high‐pressure torsion (HPT) processing. Reproduced with permission.^54^ Copyright 2017, Royal Society of Chemistry. f) The TG–differential scanning calorimetry (DSC) curve of monolayer BiO_2−_ *_x_*. Reproduced with permission.^24^ Copyright 2018, Wiley‐VCH.

ESR, positron annihilation spectroscopy (PAS), Raman spectroscopy, and XPS are also used for qualitative and quantitative characterization defects. ESR spectroscopy is particularly suitable for studying unpaired electrons in materials, and different type of defects can be easily identified according to *g* factors. For example, the *g* = 1.991, 1.998, and 2.003 corresponding to Ti^3+^, Ti vacancies, and oxygen vacancies in TiO_2_ respectively (Figures 2a and 3c). As shown in **Figure** **5**a,b, according to the qualitative relationship between vacancy and excess electron (one Ov is accompanied by one excess of electron),^79^ the defects concentration can be evaluated by the double integration of vacancy peaks in ESR spectra.^80^ Highly sophisticated pulse techniques such as 2D ^1^H TQ‐SQ MAS NMR methods has been performed to determine the defects in TiO_2_ and exclude the interference from water signals.^46^ The appeared signal at (6.00, 6.00 + 6.00 + 6.00) demonstrates the formation of Ti vacancies. PAS especially positron annihilation lifetime spectroscopy (PALS) is a mature technique to provide more accurate information, including defect types, sizes of vacancies, and defect concentrations.^81^, ^82^ For example, Liu et al. found both ultrathin CoSe_2_ nanosheets and bulk CoSe_2_ have three distinct lifetime components from its positron lifetime spectra (τ1, τ2, and τ3), and they are attributed to positron annihilation trapped by metal vacancies, defect clusters, and the interface presented in the material (Figure 5c,d).^82^ In addition, the defects concentration can be obtained by fitting the relative intensity of PALS. Raman spectroscopy provides a structural fingerprint by which molecules can be recognized chemically and chemical bonds can be identified. The presence of defects in catalyst changed the vibrational mode because different group states and chemical bonds have different vibration modes, causing the shift, appear or disappear of Raman peak. For example, oxygen vacancies in ZnO show new Raman peak at about 577 cm^−1^ (Figure 5e).^54^ On the other hand, the relative defects concentration can be evaluated by the Raman signal intensities. Gao et al. synthesized oxygen vacancies CeO_2_ and found the Raman band at about 455 and 600 cm^−1^ are ascribed to the fluorite‐type structure and intrinsic oxygen vacancies.^83^ The larger the ratio of two peak areas (A600/A455 in Raman spectra) means the higher the oxygen deficiency concentration. XPS is a surface‐sensitive, quantitative spectroscopic technique that can be used to analyze surface chemistry, including catalyst defects or elemental electronic states. The presence of defects can lead to changes in its binding energy due to the change in electron cloud density of atoms around the defect sites, which can shift in the direction of higher or lower binding energy (Figure 2b,c). By combining XPS and ESR spectra results, one can distinguish the surface and bulk oxygen defects.^80^ Thermogravimetric (TG) measurement can be used to quantitatively estimate the concentration of oxygen defects in catalyst. For example, from the TG curve of BiO_2−_ *_x_* nanoplates in Figure 5f, the surface adsorption water loss leads to the weight loss at the temperature of 100–260 °C, and at 260–400 °C the weight loss is due to the oxygen released by reducing Bi (V) to Bi (III) (BiO_2−_ *_x_* → Bi_2_O_3_ + O_2_).^24^ The amount of Bi_2_O_3_ and the released O_2_ can be obtained, and then *x* can be calculated in BiO_2−_ *_x_*. Additionally, aberration‐corrected transmission electron microscopy (ACTEM) is applied to directly observe defects structure of MoS_2_ and quantify sulphur vacancies concentration.^84^

## 4 Effect of Defect Structure on Photocatalytic Water Splitting

Photocatalytic water splitting involves light harvesting, carrier generation, carrier migration or recombination, and surface redox reactions.^10^, ^85^, ^86^, ^87^ To achieve high photocatalytic quantum efficiency, all the aspects should be considered. Defects distribution including surface and bulk vacancies, and defects concentration play different role in this process.

### 4.1 Influences of Defects Distribution

**Figure 6:**
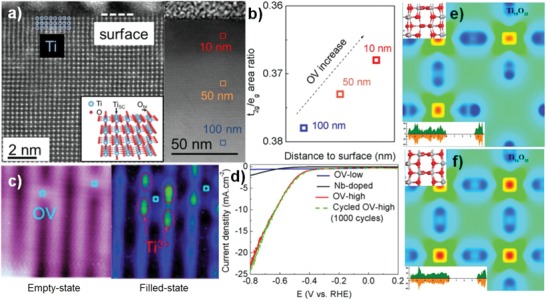
a) Cross‐sectional scanning transmission electron microscopy (STEM) and large‐region cross‐sectional STEM image of reduced TiO_2_(110) single crystal in HAADF mode. b) OV concentration from the inner bulk region to the surface region. c) STM image of the reduced TiO_2_ surface with two individual OVs in the empty state (1.2 V, 20 pA) and Ti^3+^ ions in the filled state (−2.3 V, 10 pA). d) Linear sweep voltammetric (LSV) data on different electrocatalysts for the HER at the rate of 10 mV s^−1^. Reproduced with permission.^59^ Copyright 2018, American Chemical Society. e,f) Charge density difference of Ti‐defected and normal TiO_2_. Reproduced with permission.^7^ Copyright 2015, American Chemical Society.

Surface and bulk defects play different role in photocatalytic water splitting although its exact role is still unclear.^29^, ^81^, ^88^, ^89^ Initially, both surface and bulk defects are considered as photogenerated carriers trapping and recombination centers, which results in reduced photocatalytic activity.^3^ Subsequent research found surface anion vacancies are conducive to improve the photocatalytic efficiency. Kong et al. reported that increasing ratio of surface defects to bulk defects can increase the photocatalytic activity due to the enhanced charge separation efficiency.^50^ Feng et al. found that the enhanced conductivity and electron transfer is attributed to subsurface oxygen vacancies (mainly in the 50 nm area close to the surface) and low‐coordinated Ti^3+^ in TiO_2_ (**Figure** **6**c,d).^59^ Liu et al. also confirmed that subsurface defects TiO_2_ presents high photocatalytic activity and stability through theoretical calculation.^66^ Subsurface oxygen defects in TiO_2_ are inert toward reactive oxygen species and surface oxygen defects sites are easily repaired. Furthermore, the calculated formation energy of subsurface oxygen vacancies is lower than that of surface oxygen vacancies. By contrast, cation vacancies are shallow electron acceptors, which can change structure characteristics and induce many novel physiochemical properties. Usually, cation vacancies switch the conductivity from n‐type to p‐type and accelerate the separation of photogenerated carriers. Theoretical calculation also confirmed that cation vacancies change the charge density and valence band edge of metal‐based photocatalysts (Figure 6e,f).^7^, ^20^ The band structure of photocatalyst can be regulated by defects position. For example, Zhang et al. found the presence of surface and bulk defects in TiO_2_ change the conduction band minimum, but not change the valence band maximum.^28^

Bulk defects can capture photogenerated carriers easily and are difficult to release them.^48^ In the bulk phase of photocatalyst, anion vacancies often act as trap states and recombination centers for carriers, which results in electronic localization, low reactivity, and decreased photocatalytic efficiency. Zhu et al. found that BiPO_4_ bulk defects with bismuth and oxygen vacancies strictly inhibit the separation of carriers and reduce the photocatalytic activity.^48^ Zhang et al. found that the surface and subsurface bismuth and oxygen defects of BiOCl promote photocatalytic water splitting, but the bulk‐phase oxygen vacancies play negative role.^35^ However, some studies implied that surface and bulk defects play a synergistic role in photocatalytic processes. Zuo et al. synthesized self‐doped bulk Ti^3+^ TiO_2_ by a simple one‐step combustion method, and obtained high photocatalytic hydrogen production.^90^ Additionally, the self‐doped bulk Ti^3+^ TiO_2_ photocatalyst is highly stable in air or water and can be recycled. Zhang et al. demonstrated the effect of surface/bulk oxygen defects in TiO_2_ on band structure and photocatalytic hydrogen evolution activity.^28^ They found both surface and bulk oxygen defects can promote light absorption, accelerate electrons–holes separation, and increase the donor density. But bulk oxygen defects in TiO_2_ induce a band tail below the redox potential of H^+^/H_2_, which inhibits hydrogen production. Wang et al. found that bulk oxygen vacancies WO_3_ can improve the activity of photocatalytic water oxidation by narrowing the bandgap to broaden the harvesting of visible light and restrain photogenerated carrier recombination slightly. But surface oxygen vacancies have far important role in promoting photocatalytic water oxidation activity.^29^ Our group also found the subsurface and even bulk oxygen defects in CeO_2_ are beneficial to improve photocatalytic water oxidation activity, as confirmed by DFT calculation.^5^ Particularly, when oxygen vacancies are only distributed in the bulk phase, they are unstable unless surface defects are saturated.^91^, ^92^ Meanwhile, the surface oxygen defect can be repaired by adsorption water or oxygen, therefore these defects are instable. **Table** **2** summarizes recent result on how the defect distributions affect the performance of photocatalyst, which shows usually surface defects play more critical role in enhancing catalytic activity. However, literatures show many contrary opinions, and more detailed studies are needed.

**Table 2:** Summarized opinions on role of surface, subsurface and bulk defects in photocatalytic, electrocatalytic, and other reactions

Photocatalysts	Defect type	Defect position	Role of defect in reaction	Reactions	Ref.
TiO_2_	Oxygen vacancy	Subsurface	Enhancing conductivity, promoting electron transfer, and hydrogen desorption	Electrocatalytic H_2_ evolution	59
TiO_2_	Oxygen vacancy	Surface and bulk	Decreasing bulk/surface defects ratio promote e^−^/h^+^ separation, bulk defects inducing charge recombination	Photocatalytic benzene oxidation	50
TiO_2_	Oxygen vacancy	Surface	Promoting N_2_ adsorption and activation	Photocatalytic fixation of nitrogen	56
TiO_2_	Oxygen vacancy	Surface/subsurface and bulk	Surface/subsurface defects enhance charge‐carrier separation, bulk defects induce charge recombination	Photocatalytic H_2_ evolution	89
TiO_2_	Oxygen vacancy	Surface and bulk	Promoting separation of electron–hole pairs, enhancing light absorption, increasing donor density	Photocatalytic H_2_ evolution	28
TiO_2_	Titanium vacancies	Surface and bulk	Inducing p‐type conductivity, room‐temperature ferromagnetism, more efficient charge separation and transfer	Photocatalytic H_2_ evolution and organic degradations	7
TiO_2_	Oxygen vacancy	Surface and subsurface	Subsurface defects improve light‐harvesting and facilitate charge separation, ensuring stability of surface catalytic sites	Theoretical calculation oxygen evolution	66
MoS_2_	Sulfur vacancy	Surface	As new catalytic sites, regulating hydrogen adsorption free energy, improving catalytic activity	Electrocatalytic H_2_ evolution	84
ZnS	Zinc vacancy	Surface	Modifying electronic structure, inhibiting photocorrsion, promoting charge separation and electrons transfer	Photocatalytic H_2_ evolution	40
WO_3_	Oxygen and tungsten vacancies	Surface	Facilitating interface charge transfer, improving conductivity	Photo‐electrochemical water oxidation	47
WO_3_	Oxygen vacancy	Surface	Appropriate concentration oxygen defects suppress the recombination of photoinduced carriers.	Photocatalytic oxygen evolution	96
WO_3_	Oxygen vacancy	Surface and bulk	Both surface and bulk oxygen vacancies promote the activity, surface vacancies play more role than bulk vacancies	Photocatalytic water oxidation	29
Co_3_O_4_	Oxygen vacancy	Surface	Creating more active sites, improving electronic conductivity	Electrocatalytic oxygen evolution	63
ZnO	Oxygen vacancy	Surface	Enhancing activation of CO_2_	CO_2_ electrochemical reduction	97
SrTiO_3_	Oxygen vacancy	Surface	Appropriate concentration oxygen defects improve photocatalytic performance	Photocatalytic H_2_ evolution	98
BiOBr	Oxygen vacancy	Bulk	Excitons can be dissociated into charge carriers with the incorporation of oxygen vacancy	Photocatalytic superoxide radical generation, selective oxidative‐coupling reaction	99
BiOCl	Bismuth and oxygen vacancies	Surface/subsurface and bulk	Surface/subsurface defects narrow bandgap and as active sites, promoting charge‐carrier separation, bulk defects introduce carrier trapping and as recombination sites	Photocatalytic water splitting	35
BiOCl	Oxygen vacancy	Surface	Activating H_2_O, facilitating water oxidation	Photocatalytic water oxidation	32
BiPO_4_	Bismuth and oxygen vacancies	Bulk	Inhibiting the separation of photogenerated charges, reducing photocatalytic activity	Photocatalytic degradation of pollutants	48
NiCo_2_O_4_	Oxygen vacancy	Surface	Lowering adsorption energy of H_2_O and increasing OER efficiency	Electrocatalytic water oxidation	70
SnCoFe perovskite hydroxide	Tin vacancy	Surface	Exposing more CoFe active sites, modulating conductivity, increasing OER performance	Electrocatalytic oxygen evolution	69
Bi_6_S_2_O_15_	Bismuth vacancy	Surface and bulk	Improving light response ability and bandgap narrowing, promoting separation of photoinduced electron–hole pairs, bulk defects acting as recombination center of electron–hole pairs	Photocatalytic methylene blue decomposition	100

On the other hand, the defects in semiconductors can optimize the contact interface, which facilitates the formation of a direct Z‐scheme junction to maintain stronger redox capability of photogenerated carriers.^93^ For example, oxygen‐defected CdWO_4_ and CdS can form direct Z‐scheme structure instead of type II structure of CdWO_4_/CdS, which presents high activity of photocatalytic hydrogen evolution due to the enhancement of light absorption and inhibition of photogenerated charge carriers recombination, and moreover optimized redox potential.^94^ Without oxygen vacancies in CdWO_4_, the formed type II CdWO_4_/CdS shows no photocatalytic hydrogen evolution activity due to low reduction potential. Similarly, Z‐scheme WO_3_ *_−x_*/TiO_2_ composite also shows high photoactivity of hydrogen evolution.^95^ The oxygen defects in WO_3_ change the ohmic contact between WO_3−_ *_x_* and TiO_2_ and thus switch the carriers transfer pathway from type II to Z‐scheme heterojunction.

### 4.2 Influences of Defects Concentration

Defects concentration in defective photocatalysts has great influence on the photocatalytic performances. A general rule is that the more anion vacancies, the higher the electron donor density. Some studies have confirmed that TiO_2_ bandgap is dependent on the Ti vacancies concentration.^101^ Hao et al. found that excessive zinc vacancies in ZnS act as charge carrier recombination centers, which is unfavorable for photocatalytic hydrogen evolution.^40^ WO_3_ with high surface vacancies and suitable bulk vacancies has high photocatalytic water oxidation activity. The surface oxygen vacancies of WO_3_ are more effective for the separation of charge carriers than bulk oxygen vacancies, and actually charge carriers separation is critical in photocatalysis.^29^ In many cases, an appropriate concentration of surface defects can lead to improved charge separation, which ensures high activity and stability.^71^ Excessive and uncontrollable defects formation act as the recombination sites, unfavorable for photocatalytic activity.^50^

## 5 Theoretical Exploration on Defects‐Activity Relationship

Numerous experimental studies have been performed to demonstrate the effect of defects in photocatalytic water splitting, however, the mechanism remains unclear, let alone defects‐activity relationship. Combining experimental and theoretical studies can help design new catalysts and improve catalytic efficiency. In this section, we discuss the defects‐activity relationship from a theoretical perspective.

### 5.1 Water Splitting Mechanism

**Figure 7:**
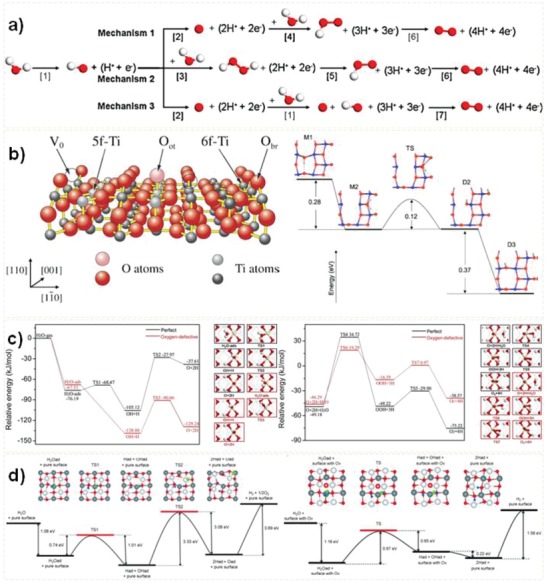
a) Three oxygen evolution reaction mechanisms on g‐C_3_N_4_. Reproduced with permission.^104^ Copyright 2015, Elsevier. b) TiO_2_ (101) model and potential energy diagram for the water dissociation pathway. Reproduced with permission.^105^ Copyright 2003, American Institute of Physics. c) Potential energy profiles for water dehydrogenation and O–O bond formation. Reproduced with permission.^5^ Copyright 2018, Elsevier. d) Overall water splitting reaction on pure BiOCl (001) surface and surface oxygen vacancy BiOCl (001) surface. Reproduced with permission.^35^ Copyright 2015, Wiley‐VCH.

The surface reaction is critical in photocatalytic water splitting.^5^, ^102^ Water adsorption and activation on model catalysts surface is the first and key step to understand the whole water splitting reaction.^82^ Pt is widely used as a cocatalyst in photocatalytic water splitting.^103^ Bazhenov et al. illustrated the mechanism of water adsorption and activation on Pt and Ru by using nanoparticle cluster models, and found that the surface active sites including coordination environment and metal d‐band center value affect the binding ability of water on metal surface, and the activation energy of water dissociation shows a linear relationship with the water adsorption energy.^103^ Lin et al. systemically calculated the photocatalytic water oxidation mechanism with dispersion‐corrected methods by using g‐C_3_N_4_ catalyst.^104^ Three main pathways were considered (**Figure** **7**a), and the binding strength of OH intermediate is the key index in assessing the activity.

The presence of defects in metal‐based photocatalysts significantly affects the adsorption energies of some species, low the activation energies of elementary steps and change the active sites. Tilocca et al. studied water dissociation on TiO_2_‐anatase (101) by using first‐principles simulations and found that the low coordination of oxygen vacancies sites is conducive to water dissociation. They proposed an indirect reaction mechanism of water dissociation (Figure 7b).^105^ Li et al. found that subsurface oxygen vacancies in TiO_2_ anatase (101) facilitate water dissociation directly.^106^ Our group studied the mechanism of O—O bond formation on oxygen defects CeO_2_ and considered all possible water oxidation pathways.^5^ As shown in Figure 7c, the optimized pathway of water oxidation is H_2_O → OH + H → O + 2H; O + 2H + H_2_O → OOH + 3H → O_2_ + 4H. Among them, O–O bond formation in the form of OOH is the rate‐limiting step, and the presence of oxygen vacancies in CeO_2_ significantly decrease the activation energies of some elementary steps including the steps of OH + H → O + 2H and O + 2H + H_2_O → OOH. Additionally, the reverse recombination reaction of O + 2H → OH + H can be restrained due to the increased binding strength of O + 2H by presence of oxygen vacancies. Zhang et al. confirmed that the surface oxygen vacancies site of BiOCl(001) are the active site of hydrogen evolution (Figure 7d).^35^

**Figure 8:**
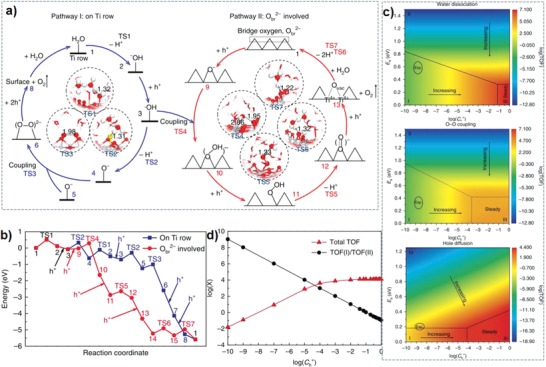
a) One of the dual pathways (pathway I) occurring on the Ti row. b) The other pathway (pathway II) involving bridge oxygen. c) Influence of different kinetic barriers (water dissociation, O–O coupling and hole diffusion) and C_h+_ on the total turnover frequency (TOF). d) The contribution ratio between pathways I and II to the total TOF. Reproduced with permission.^108^ Copyright 2018, Springer Nature.

However, most calculations are limited to the initial water activation and dissociation, using one or several water molecules,^5^, ^15^, ^107^ and very few studies focus onto the water splitting mechanism by using liquid/solid interface models, because it is complex to simulate the aqueous environment by surface radicals (trapped holes on surface) or electrons. Recently, Wang et al. developed a new multipoint averaging molecular dynamics (MPA‐MD) method to investigate the mechanism of photocatalytic water oxidation on water/TiO_2_(110) interface.^108^ They identified a dual pathway of oxygen evolution reaction (**Figure** **8**a), and discovered both pathways are easy to occur due to low barrier (Figure 8b). The radicals (·OH_t_, O_t_^−^, and O_br_^−^) play three critical roles during the surface reaction process. Frist, radicals can reduce the bonding strength of Ti‐O bonds and contribute to the desorption of O_2_. Second, radicals promote the coupling of O–O bonds. Third, radicals can effectively inhibit the recombination of photogenerated electrons and holes, and improve the photocatalytic reaction. Interestingly, based on calculation of reaction mechanism and steady‐state microkinetic analysis, they found that the low efficiency of photocatalytic oxygen evolution on TiO_2_ (110) is not due to the high energy barrier of reaction, but due to the low concentration of photogenerated holes (Figure 8c,d). They believe that the most effective way to improve the efficiency of photocatalytic water splitting is to increase the surface photogenerated holes concentration instead of reducing the energy barrier of the surface reaction. Therefore, their work confirms that increasing the concentration of photogenerated holes is the key to photocatalytic water splitting. Specially, the mass of photogenerated holes is greater than electron, and improving the diffusion ability of holes is an important strategy to promote carriers separation.

### 5.2 Understanding on Defects‐Activity Relationship

**Figure 9:**
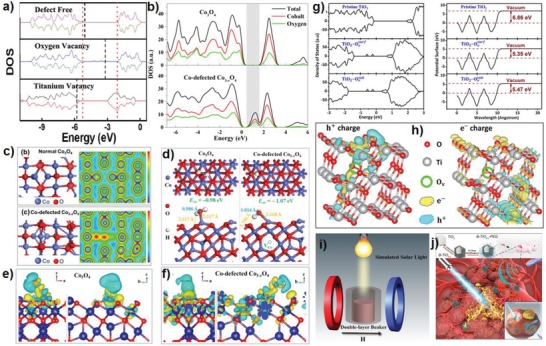
a) The DOS of perfect TiO_2_ nanosheets, oxygen vacancies TiO_2_, and titanium vacancies TiO_2_. Reproduced with permission.^109^ Copyright 2012, American Chemical Society. b) Total and projected charge density of states for Co_3_O_4_ and Co‐defected Co_3−_ *_x_*O_4_, and c) the optimized cell structures and the corresponding charge density mapping images. d) Optimized structures of water adsorbed on perfect Co_3_O_4_(111) and Co‐defected Co_3−_ *_x_*O_4_(111). e) Deformation charge density of H_2_O‐adsorbed structure of Co_3_O_4_ and f) Co‐defected Co_3−_ *_x_*O_4_. Reproduced with permission.^8^ Copyright 2018, American Chemical Society. g) DOS and potential surfaces of TiO_2_ (101) surfaces. h) Simulated charge distribution. Reproduced with permission.^66^ Copyright 2018, American Chemical Society. i) Schematic diagram of the electromagnet‐photocatalysis apparatus. Reproduced with permission.^112^ Copyright 2018, American Chemical Society. j) Schematic illustration of B‐TiO_2−_ *_x_*‐PEG for enhanced/synergistic sonodynamic and photothermal therapy of cancer cells. Reproduced with permission.^51^ Copyright 2018, American Chemical Society.

The presence of cation defects sites in photocatalyst is favorable to control the adsorption and desorption of reactants, intermediates, and products (**Figure** **9**d). When interaction between defects sites and adsorbed molecules occurs, electron‐rich vacancies sites can pull down the lowest unoccupied orbital position, and is favorable for electron transfer.^22^ From the density of states (DOS) images, the presence of cation defects can broaden the VB by upshifting VB maximum, resulting in electron delocalization (Figure 9a,b,c).^109^ A wider VB leads to higher photogenerated holes migration and better photocatalytic oxidation ability.^5^, ^7^, ^20^, ^43^ Cationic vacancies generally increase electrical conductivity and donor density as shallow acceptors. Moreover, the cation vacancies also provide more active sites for initial water adsorption and activation.^8^ Therefore, cation vacancies can enhance transfer and separation of photogenerated electrons and holes, and results in high degree of electron delocalization (Figure 9e,f), as well provides more active sites for water‐splitting surface reaction including the adsorption and activation of water and other intermediates.

On contrary, anion vacancies in metal‐based photocatalysts often act as shallow donors, which can increase the donor density in photocatalytic process.^110^ And the presence of anion vacancies can induce new electron stats or trap states with deep or shallow energy levels within the forbidden band.^111^ Mid gap states may appear due to large amounts of lattice disorder, corresponding to different energy distributions. Moreover, the mid gap states position can be effectively regulated by adjusting the distribution and concentration of vacancies.^28^ The new defect states increase the charge density around the VB maximum, improve the conductivity, and act as a transmission medium for easy separation of photogenerated holes and electrons.^97^ When the anion vacancies‐induced mid gap states overlaps with CB or VB, the bandgap is narrowed, thereby enhancing light absorption. It is worth noting that anion vacancies can also produce bandgap narrowing and mid gap states simultaneously. The anion vacancies have additional effect of producing band tailing or band bending, which not only make electrons to accumulate around the anion vacancies, but also increases the electrical conductivity and promotes surface reactions such as molecular adsorption and activation.^110^ Here we choose oxygen deficient TiO_2_ as an example. In the bulk TiO_2_, each oxygen atom binds three Ti atoms, and thus with the presence of oxygen vacancy, two residual electrons must be shared by the surrounding bonded Ti atoms. Therefore, Ti^4+^ accepts electrons to generate Ti^3+^ to regulate the electronic structure of catalyst surface, providing more active sites for the adsorption, reaction and desorption of reactants, intermediates, and products. From the potential surfaces in Figure 9g (right), the work function of perfect TiO_2_, TiO_2_ with surface oxygen vacancies and TiO_2_ with subsurface oxygen vacancies are 6.86, 5.35, and 5.47 eV, respectively.^66^ The electrons easily transfer from photocatalyst with lower work function to higher work function, which achieves the separation of carriers. Therefore, the presence of oxygen vacancies in TiO_2_ facilitates the separation of holes–electron pairs. In addition, the photogenerated holes accumulate at the oxygen defect sites, and electrons shift elsewhere apart from oxygen vacancies sites (Figure 9h). Similarly, oxygen vacancies ZnO significantly decreases the adsorption Gibbs free energies of some intermediates during electrocatalytic reduction of CO_2_ compared to that on pristine ZnO, and presents high electrocatalytic performance.^97^ In addition, the presence of oxygen vacancies reduces the effective mass of electron of oxygen defective CeO_2_, results in a high mobility of electrons.^5^ **Table** **3** summarizes the photocatalytic HER and OER performance in comparison of defected and normal catalysts, which shows defect engineering can significantly improve the activity.

**Table 3:** Summarized photocatalytic hydrogen evolution reaction (HER) and oxygen evolution reaction (OER) activity of defect‐based photocatalysts

Reaction Type	Catalysts	Sacrificial Agents	Cocatalysts	Illumination	Activity [µmol [g_cat_ h]^−1^]	Ref.
HER	Ti defected TiO_2_ Normal TiO_2_	CH_3_OH	1.0 wt% Pt	UV light	29 800 6800	7
HER	TiO_2_ with Ti and O vacancies n‐type TiO_2_	CH_3_OH	1.0 wt% Pt	UV light	50 300 6750	13
HER	O defected TiO_2_ TiO_2_ (P25)	CH_3_OH/H_2_O	1.0 wt% Pt	λ > 400 nm	115 4	113
HER	O defected TiO_2_ Normal TiO_2_	CH_3_OH/H_2_O	1.0 wt% Pt	Simulated sunlight	2139 169	114
HER	Sub‐10 nm rutile TiO_2_ Hydrogenated H‐TiO_2_ TiO_2_ P25	CH_3_OH/H_2_O	1.0 wt% Pt	λ > 400 nm	932 107 3	89
HER	Zn defected ZnS Normal ZnS	Na_2_S/Na_2_SO_3_	None	λ ≥ 420 nm	337 83	40
HER (from NH_3_BH_3_)	Cu defected Cu_2_ *_−x_*S Normal Cu_2_S	None	10 wt% Pd	λ > 420 nm	157 040 122 230	67
HER	Surface defected Zn‐Cd‐S Defect free Zn‐Cd‐S	Na_2_S/ Na_2_SO_3_	Pt	λ > 420 nm	11 400 2800	34
HER	Ti^3+^ self‐doped TiO_2_ Stoichiometric TiO_2_	CH_3_OH/H_2_O	1.0 wt% Pt	λ > 400 nm	181 <1	115
HER	O defected K_4_Nb_6_O_17_ Normal K_4_Nb_6_O_17_	CH_3_OH	None	300 W Xe lamp	1661 78	116
HER	O defected SrTiO_3_ Normal SrTiO_3_	CH_3_OH	1.0 wt% Pt	UV–vis	2200 980	98
OER	O defected CeO_2_ Normal CeO_2_	AgNO_3_	None	λ ≥ 420 nm	137 78	5
OER	O defected BiOCl (010) O defected BiOCl (001) TiO_2_ P25	None	None	UV light	100 32 0	32
OER	O defected CeO_2_ Normal CeO_2_	AgNO_3_	None	λ ≥ 420 nm	353 33	117
OER	H_2_ treated WO_3_ Normal WO_3_	AgNO_3_	None	λ ≥ 420 nm	376 163	118
OER	O defected WO_3_ Normal WO_3_	AgNO_3_	None	λ > 400 nm	120 µmol [m^2^ h]^−1^ 60 µmol [m^2^ h]^−1^	29

## 6 Summary and Challenge

Defective metal‐based catalysts are showing great potential for photocatalytic water splitting. This review summarizes the latest progress in the defective photocatalysts for water splitting. Metal‐based semiconductors with cations, anions or both cation and anion vacancies exhibit many novel properties. The light adsorption, photogenerated carrier separation and migration, and surface reaction process are promoted by proper vacancies in photocatalysts. Advanced characterization techniques combined with density functional theory calculations provide insight information to determine the structure and performance of defective photocatalysts. Despite tremendous efforts and some achievements described here, a deep and thorough understanding of defects‐activity relationship still remains huge challenge; meanwhile the development of high efficiency, superior photo stable, and low‐cost defective photocatalysts is essential.

More efforts should be addressed to overcome current problems and challenges. First, how to probe defect structure changes during photocatalytic reaction and how to characterize it? Many studies only focused on revealing the defect structure, concentration, and defect distribution of defective photocatalysts, but rarely reported the defect structure change during photocatalytic reaction and its effects for water splitting. Actually the in situ formed species, which may be the real active sites in photocatalysis, are never considered. Development of advanced in situ characterization technology is helpful to understand the defects change during photocatalysis at the atomic level. Second, how to solve the poor stability of defective metal‐based photocatalysts? Effective and controllable methods are needed to synthesize stable vacancies‐rich catalysts. Third, how to design high‐visible or even near‐infrared (more than about 50% of solar radiant energy) absorption catalyst with high charge separation efficiency by defect engineering is beneficial for applications. A characteristic of oxygen‐defective semiconductor is the absorption peak in visible light region, however it is not clear whether this absorption can benefit the reaction. Fourth, there is a crucial need to understand the relationship between defects and reactivity, the mechanism of water splitting in a real aqueous environment to eventually achieve the results of theoretical calculations and to guide experimental efforts. Therefore, it is an urgent need to develop more advanced DFT software with precise simulation experimental conditions.

Therefore, future researches are suggested to be directed in the following fields. First, it is necessary to develop new methods for precise and controlled synthesis of defect‐rich photocatalysts with high activity and high stability. Second, more advanced in situ characterization techniques and theoretical calculation are vital. Third, combining defective catalysts with a second component to form Z‐scheme junction is promising, which would be increases the water splitting efficiency. Fourth, designing additional external driving force enhances the separation ability of photogenerated carriers. For example, magnetic field boost carrier transport (Figure 9i)^112^ and the application of piezoelectric materials. Investigation of defective metal‐based catalysts for new applications, such as medical field (Figure 9j)^51^ and other catalysis like hydrogenation^26^, ^27^, ^119^ and electrocatalysis et al^120^, ^121^, ^122^ will deep understanding on structure‐activity relationship of defects, which is in turn helpful for exploring defect‐based photocatalyst.

## Conflict of Interest

The authors declare no conflict of interest.
